# Intermittent Theta Burst Over M1 May Increase Peak Power of a Wingate Anaerobic Test and Prevent the Reduction of Voluntary Activation Measured with Transcranial Magnetic Stimulation

**DOI:** 10.3389/fnbeh.2016.00150

**Published:** 2016-07-19

**Authors:** Louis-Solal Giboin, Patrick Thumm, Raphael Bertschinger, Markus Gruber

**Affiliations:** Sensorimotor Performance Lab, Sport Science Department, Universität KonstanzKonstanz, Germany

**Keywords:** rTMS, supraspinal fatigue, central fatigue, performance, cycling, muscle fatigue, power, iTBS

## Abstract

Despite the potential of repetitive transcranial magnetic stimulation (rTMS) to improve performances in patients suffering from motor neuronal afflictions, its effect on motor performance enhancement in healthy subjects during a specific sport task is still unknown. We hypothesized that after an intermittent theta burst (iTBS) treatment, performance during the Wingate Anaerobic Test (WAnT) will increase and supraspinal fatigue following the exercise will be lower in comparison to a control treatment. Ten subjects participated in two randomized experiments consisting of a WAnT 5 min after either an iTBS or a control treatment. We determined voluntary activation (VA) of the right knee extensors with TMS (VA_TMS_) and with peripheral nerve stimulation (VA_PNS_) of the femoral nerve, before and after the WAnT. *T*-tests were applied to the WAnT results and a two way within subject ANOVA was applied to VA results. The iTBS treatment increased the peak power and the maximum pedalling cadence and suppressed the reduction of VA_TMS_ following the WAnT compared to the control treatment. No behavioral changes related to fatigue (mean power and fatigue index) were observed. These results indicate for the first time that iTBS could be used as a potential intervention to improve anaerobic performance in a sport specific task.

## Introduction

Repetitive transcranial magnetic stimulation (rTMS) may be able to induce transient plasticity in cortical neural networks and thus can modify corticospinal excitability as well (Ziemann, 2004; Hoogendam et al., 2010). Its application over the primary motor cortex (M1) has been widely used as a therapeutic tool for many conditions with various levels of success (Ziemann, 2005; Ridding and Rothwell, 2007; Lefaucheur et al., 2014; Palm et al., 2014). Several rTMS protocols were applied to patients suffering from a diversity of motor neuronal disorders, which consistently improved motor performance or even led to the recovery of motor function (Lefaucheur et al., 2004; Khedr et al., 2006; Kakuda et al., 2013; Tretriluxana et al., 2013; Yang et al., 2013). In healthy subjects, applications of rTMS protocols have been tested more sparsely, and only a few studies looked at performance in sport specific tasks (Muellbacher et al., 2000; Schlaghecken et al., 2003; Carey et al., 2006; Hortobagyi et al., 2009; Ward et al., 2010; Censor and Cohen, 2011; Teo et al., 2011). In a fundamental study, Benwell et al. (2006) examined the impact of rTMS in a maximal performance task. The authors were able to demonstrate that during a continuing maximal isometric voluntary pinch grip, the rate of force loss was lower over time after the paired-pulse rTMS intervention, indicating a possible effect of this intervention on muscle fatigue. These results indicated that the transient increase in cortical excitability after the rTMS protocol can, at least in part, compensate the loss of neural drive induced by central fatigue, i.e., “a progressive reduction of voluntary activation (VA) of muscle during exercise” (Gandevia, 2001).

This view has been further strengthened by several studies showing a positive effect of anodal transcranial direct current stimulation on different strength tasks (Cogiamanian et al., 2007; Tanaka et al., 2009; Williams et al., 2013). However, although the potential effects of non-invasive brain stimulation on central fatigue have been derived from behavioral studies, this has not yet been demonstrated directly with the twitch interpolated technique (Benwell et al., 2006; Cogiamanian et al., 2007). Moreover, the tasks performed have been single joint “laboratory tasks” and therefore the results cannot be directly transferred to “real world” multi-joint sport tasks.

The Wingate anaerobic test (WAnT) is a high intensity anaerobic sport specific test that seems to be ideally suited to examine performance and fatigue related modulations after rTMS (Coppin et al., 2012). Indeed, it has been shown that the WAnT is able to reduce drastically the force production of a following maximal isometric knee extension, and that this force reduction was associated with peripheral and central mechanisms (Fernandez-del-Olmo et al., 2013). Based on the impact of one WAnT on the another one, which was done 30 min later, the authors concluded that central rather than peripheral mechanisms might be responsible for a reduction in performance occurring after the WAnT (Fernandez-del-Olmo et al., 2013).

In the present study we hypothesized that an increased corticospinal excitability induced by rTMS will increase neural drive and performance in a sport specific task. Thus, we expected to find a higher power output during, and a higher resistance to central fatigue after the WAnT. For the rTMS treatment, we used the intermittent theta burst (iTBS) protocol, which was first described by Huang et al. (2005). To examine the potential effect of iTBS on central fatigue, we estimated the VA with TMS and peripheral nerve stimulation (PNS) in the quadriceps before and after the WAnT in two different sessions; one after real iTBS, the other after sham iTBS.

## Materials and Methods

### Subjects

Ten regularly active males (mean/SD; age: 26 years, SD 2 years; weight: 78 kg, SD 6 kg; height: 181 cm, SD 4 cm) participated in the study, after giving written informed consent. The Ethics Committee at Konstanz Universität approved the study procedure. One part of the protocol was that subjects were deliberately kept naive about the existence of a placebo treatment. Contrariwise, they were informed that in the on going experiment two different treatment protocols were being tested. So, they were able to experience the difference between the protocols but throughout the whole study they were not aware of the existence of a control treatment.

### General Protocol

**Figure 1:**
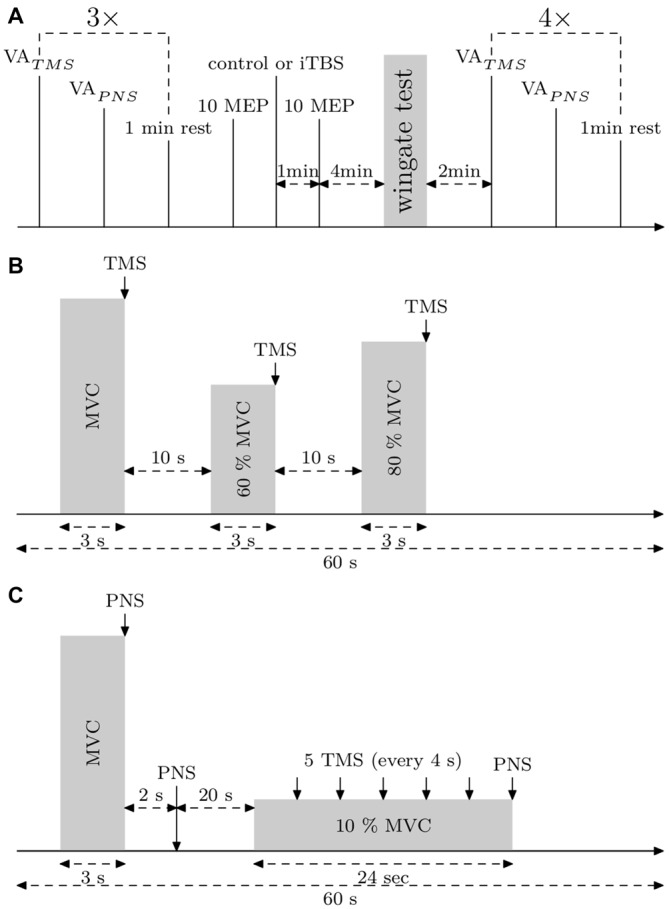
**Experimental protocols. (A)** General protocol. Voluntary action (VA)_TMS_, VA_PNS_ and motor evoked potentials (MEPs) (10% maximal voluntary contraction [MVC]) were measured three times. Then, 10 MEPs (10% MVC) were applied to control the basal corticospinal excitability. After that, the control or the intermittent theta burst (iTBS) treatment was carried out and 1 min later, 10 MEPs (10% MVC) were applied. Then, the subject warmed up 3 min before the Wingate Anaerobic Test (WAnT). Immediately after the WAnT, the subject was fixed in the measurement chair and VA_TMS_, VA_PNS_ and MEPs at 10% MVC were measured four times. **(B)** Protocol describing VA_TMS_ measurements. **(C)** Protocol describing VA_PNS_ measurements and MEPs (10% MVC) measurements.

Subjects participated in three familiarizations sessions and then in two experimental sessions consisting of a WAnT that was performed 5 min after either an iTBS or a control treatment. The experiment was conducted in counter balanced randomized order, i.e., five subjects started with the control treatment and five subjects with the iTBS treatment. VA_TMS_, VA_PNS_, muscle twitch size at rest and motor evoked potentials (MEPs) of the right vastus lateralis (VL) were measured three times prior to the iTBS treatment and four times after the WAnT (measurement starting 2 min after the end of the WAnT, see Figure 1A). Both sessions were separated by at least 2 (and at the most, 7) days of recovery. During neurophysiological measurements the subject was seated in a custom made chair with adjustable height and depth.

### Familiarization Sessions

Prior to the two experimental sessions, the subjects participated in three familiarization sessions to get used to the WAnT, maximal voluntary contraction (MVC), TMS and PNS measurements. The three WAnTs reduced the bias induced by a possible training effect in the main study. At least 2 days and a maximum of 7 days separated each familiarization sessions.

### Wingate Anaerobic Test

The WAnT was performed on a Cyclus2 ergometer (RBM elektronik-automation GmbH, Leipzig, Germany). Just before each WAnT, a warm-up of 3 min at an intensity of 1 W/kg body weight (BW) and a self-selected cadence was performed, interspersed with a seated sprint lasting 3–4 s that was started at the first and second minute of the warm-up. The WAnT was programmed for duration of 30 s, starting at a cadence of 80 rpm, applying a constant force to the cranks automatically. Thus, the subjects were instructed to pedal at 60 rpm, and when they felt ready, to accelerate as fast as possible to the highest attainable frequency and to maintain it throughout the whole test duration while remaining in a seated position. The investigators ensured that the subject’s motivation was maximal and gave strong verbal encouragement throughout the 30 s test duration.

The resistive load (simulated flywheel mass) was individually adjusted using the following equation: Load = (Bodyweight)^2^/1000. Mechanical data was sampled at a frequency of 8 Hz. The power of the ergometer was calculated by the cyclus2 software as followed: *P* = 2 * Pi * M/Tr where M is the torque at rear wheel in Nm and Tr the time for one rear wheel revolution in second. The overall error is SD 0.25% in the power range of 100–2000 W. Peak power corresponds to the highest power output achieved during the 30 s test. Maximum cadence corresponds to the maximum pedalling rate reached during the 30 s test. Time-to-peak corresponds to the time needed to reach peak power, starting from the time point where a cadence of 80 rpm was exceeded. Mean power is the average power calculated for the complete test duration. We calculated the fatigue index as the average decline in power per second from the time point where peak power was reached until the end of the test.

### EMG

Bipolar surface electrodes (Bagnoli DE-2.1, DELSYS, Natick MA, USA; interelectrode distance: 10 mm, electrode size: 1 × 10 mm) were applied over the muscle belly of the right VL, rectus femoris, vastus medialis and biceps femoris (BF) in the direction of the underlying muscle fibers. Beforehand, we shaved, sanded, and cleaned the skin with alcohol and placed the reference electrode over the right acromion. EMG signals were filtered (bandpass between 20 and 450 Hz), amplified (*1k), sampled (4000 Hz) and registered on a computer with an analog-digital board (Micro 1401, CED, Cambridge Electronic Design Limited, Cambridge, England) for a posteriori analysis with Signal Software (CED Limited, Cambridge, England).

### Force Recordings

The subjects were positioned in the chair with 90° knee and hip angles. A non-compliant tension belt was fixed ~2 cm above the right lateral malleolus (location was drawn on the skin for an exact repositioning pre and post WAnT) and connected to a force transducer (Model 9321A, Kistler, Winterthur, Switzerland) attached under the chair. Force signals from isometric quadriceps contractions were amplified and stored together with EMG data. A visual feedback of the current strength was displayed on a screen, with variation of scaling during the experiment and with the investigator indicating a target to reach (10–20% over the MVC) as a supplementary motivational factor. During every contraction, the subject crossed his arms in front of his chest and was instructed to keep this position. The Investigator ensured that the subject’s motivation was maximal during the whole study and gave a strong verbal encouragement throughout the MVC trials.

### Peripheral Nerve Stimulation (PNS)

The cathode (custom made, 5 cm^2^, copper) was fixed on the femoral nerve in the femoral triangle and the anode (custom made, 24 cm^2^, bendable copper) on the center of the gluteus maximus. With this placement of the electrodes, PNS induced M-waves in the quadriceps without any muscular activity in the EMG of the BF. The locations of the electrodes were drawn on the skin for an exact replacement pre- and post-WAnT.

A Digitimer DS7A stimulator (Welwyn Garden City, UK) delivered single electrical stimuli (1 ms duration). The stimulus intensity to evoke Mmax was determined at rest (stimulation intensities required to reach Mmax were done between 20 and 60 mA depending on the subject). PNS intensity was set at 130% Mmax for supramaximal stimulations that were delivered in order to determine VA.

### Transcranial Magnetic Stimulation (TMS)

Biphasic TMS pulses were delivered by a figure-of-eight coil, specifically designed to stimulate lower limb motor cortical area (MC-B70, MagVenture), and produced by a MagPro R30 Stimulator (MagVenture). During the whole experiment, pulses were biphasic with the current flowing in the coil in an anterior-posterior/posterior-anterior direction. The coil was held so that the figure-of-eight was oriented perpendicularly to the interhemispheric fissure, with the center of the coil placed over the left hemisphere a few centimeters lateral of the vertex. The position was adjusted in order to elicit the biggest MEP possible in VL (around 40% Mmax amplitude). The position of the coil was drawn on a swimming cap to ensure an identical position of the coil throughout the whole experiment. TMS intensity was set to elicit the biggest MEP possible during VA_TMS_ measurements (90–100% maximal stimulator output (MSO), identical intensity during the whole experiment) and a second intensity was used to produce MEPs equal to 10–20% Mmax amplitude during 10% MVC. The intensity was then kept constant during the whole experiment.

### Control and iTBS Treatment

**Figure 2:**
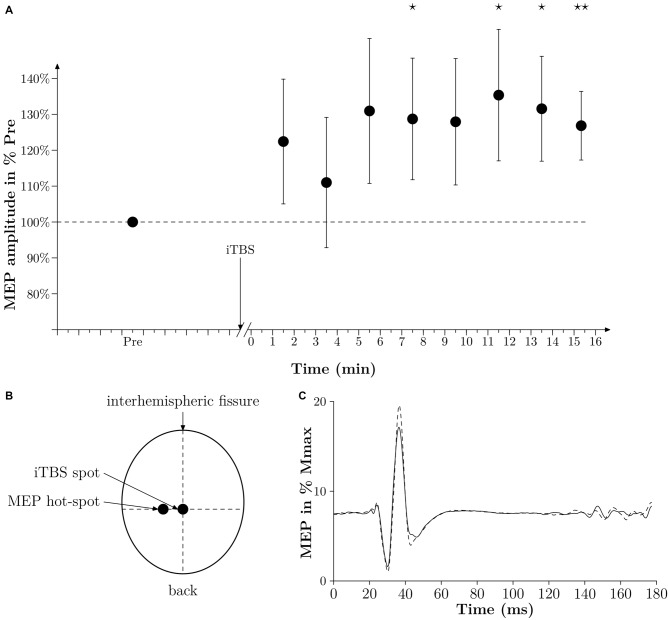
**Time effect of iTBS. (A)** Averaged time effect of iTBS in six subjects. MEPs amplitudes done at 10% MVC are expressed as a percentage of the averaged amplitude of MEPs obtained in PRE and are plotted against time (min). Thin lines represent standard error of the mean. A star represents a *p*-value < 0.05, two stars represent a *p*-value < 0.01. **(B)** Drawing of the position of the coil of the TMS to induce a MEP in the vastus lateralis (VL) (MEP hot-spot) and when applying iTBS (iTBS spot). The legend “back” indicates the back of the head. **(C)** Averaged traces of 10 PRE MEPs (thin black line) and 10 POST MEPs (dashed line) obtained 7 min after iTBS in one subject and expressed in % Mmax. Time 0 corresponds to the time of stimulation.

Because the WAnT is an extremely demanding test, we have carefully chosen the iTBS treatment as a short and comfortable rTMS protocol (Cárdenas-Morales et al., 2010). The iTBS treatment we used was identical to the one described by Huang et al. (2005) with a stimulation intensity equal to 80% of active motor threshold obtained in the VL at 20% MVC. The control treatment was identical to the iTBS treatment but with a maximal stimulation output of 2%, which was enough to produce a clicking sound but definitely too low to induce any neural plasticity. During the control and iTBS treatment, the coil was placed over the intersection point between the interhemispheric fissure and the line passing through the hot spot and perpendicular to the interhemispheric fissure (see Figure 2B). The rationale behind this placement was to modify the excitability of the leg cortical area of both hemispheres, in order to change motor output in both legs. We used this protocol because the sequential application of iTBS can affect the excitability of the contralateral hemisphere (Suppa et al., 2008), which could invert the excitability of the second iTBS (Müller-Dahlhaus and Ziemann, 2015). To control whether iTBS applied over the vertex have spreading effects over the leg cortical areas, we tested corticospinal excitability by stimulating the VL hot-spot with TMS. Thus, 10 MEPs at 10% MVC were measured a few minutes before and 1 min after the treatment. The average amplitude of the MEPs post-treatment was expressed in percentage of the average amplitude of the MEP pre-treatment. Immediately after the MEPs, the subject started to warm-up for the WAnT (see Figure 1A).

### Voluntary Activation

VA, can be estimated by comparing the amplitude of a muscular twitch during a MVC caused by a supramaximal PNS or TMS over the motor cortex (superimposed twitch), with, the amplitude of the muscular twitch caused by the same stimulus in the potentiated muscle at rest (Gandevia et al., 1995) or the amplitude of the estimated twitch at rest (Todd et al., 2004). The measure of VA through PNS (VA_PNS_) and VA through TMS (VA_TMS_) are used to demonstrate the existence of central and supraspinal fatigue respectively. Gandevia (2001) defined central fatigue as a “reduction in VA of muscle during exercise” and supraspinal fatigue as “fatigue produced by failure to generate output from the motor cortex”. Here, supraspinal fatigue is considered as a subset of central fatigue and although they cannot be directly compared mainly due to methodological restrictions (e.g., activation of different muscles during TMS compared to PNS), they can give an insight regarding the possible location of fatigue (Todd et al., 2003).

Before VA measurements, the subject warmed up 10 min with incremental isometric contractions. During VA measurements, when there was no visible plateau in MVC, or when the timing of the stimulation was not correct, or if the subject revealed it was not an MVC, the trial was rejected and repeated after 20–30 s. This case was seen only for two subjects during the pre measurements, for whom, maybe, the preceding warm-up was not enough, hence one or two more trials to reach real MVC and real VA. Stimulations were triggered manually during the plateau of the MVC. To assess VA_TMS_ we used a protocol according to Todd et al. (2004) and Sidhu et al. (2009). The estimated twitch at rest is estimated from a linear regression between the size of the superimposed twitches obtained during MVC, 60 and 80% MVC contractions (see Figure 1B) and the level of voluntary contraction. VA_TMS_ was defined as: VA_TMS_ = (1 − superimposed twitch/estimated twitch at rest) × 100.

**Figure 3:**
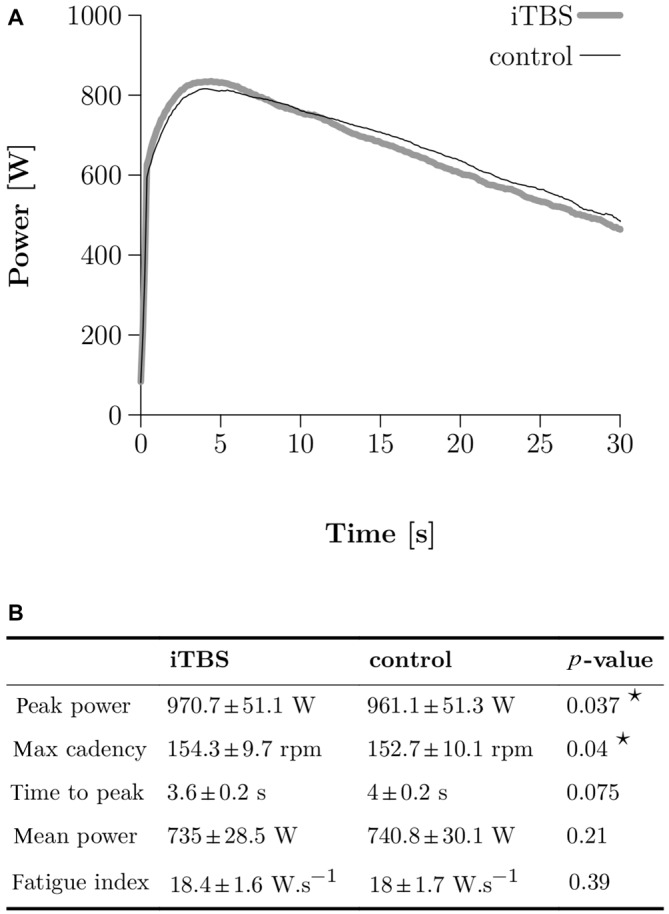
**Wingate Anaerobic Test (WAnT) results. (A)** Two typical WAnT power curves obtained in one single subject after control (thin black line) and iTBS treatment (thick gray line). **(B)** Group data (*N* = 10) for the most important parameters, which characterize the power development in the WAnT. Bilateral paired *t*-tests were applied and a star corresponds to a significant result (*p* < 0.05).

VA_PNS_ was defined as: VA_PNS_ (1 − superimposed twitch/resting twitch) × 100, where the superimposed twitch is elicited with a supramaximal PNS during MVC and the resting twitch by PNS, 2 s after the MVC (see Figure 1C). VA_PNS_ was measured 1 min after VA_TMS_ and there was 1 min of rest before the next cycle (see Figure 1A). The best out of the 3 VA measurements performed before the WAnT was taken as the pre WAnT VA value (as depicted in Figure 3).

### Corticospinal Excitability

To assess corticospinal excitability, 5 MEPs at 10% MVC (with interstimulus intervals set at 4 s) were measured starting 20 s after VA_PNS_ (see Figure 1C). The amplitude of each MEP was normalized to the amplitude of the following Mmax elicited at 10% MVC.

### Maximal Voluntary Contraction Before and After the Task

We measured the maximal force (MVC) during the VA_TMS_ measurements pre- and post-WAnT, with and without iTBS, and expressed the MVC in Newtons.

### Time Course Effect of iTBS on Corticospinal Excitability

We performed an additional experiment on six subjects to verify that the iTBS protocol we used in the present study was able to increase the corticospinal excitability substantially (see Figure 2A). In this additional experiment we modified the time delay from the end of the iTBS treatment and the TMS measurements. With this protocol we were then able to follow the changes in MEPs from 1 min after iTBS until 15 min after iTBS. At the beginning of the experiment, the subject had to warm up with isometric contractions. MVC was assessed after three maximum trials separated by 1 min of rest. The intensity of TMS was set so the MEP amplitude during a contraction of 10% MVC was equal to 10–20% of the Mmax amplitude, which had already been determined at 10% MVC beforehand. One set of MEPs consisted of 10 MEPs induced every 4 s. One set was done 5 min before iTBS (PRE), and then 1 min after sets were done every 1 min 20 s for 15 min. MEP amplitudes were expressed in percentages of the averaged MEP amplitude obtained in PRE.

### Statistical Analysis

All statistical tests were done with R (3.1.0, copyright 2014, The R Foundation for Statistical Computing Platform). Two-tailed paired* t*-tests were conducted to compare peak power, time-to-peak, mean power, maximum cadence, fatigue index, and pre- and post-treatment MEP amplitude. Unilateral one sample *t*-tests were conducted on MEP amplitudes post-iTBS (time course effect of iTBS experiment) to show a possible difference with a value of 100 (i.e., PRE value). Two ways within subject ANOVAs were applied for muscle twitch size at rest, VA_PNS_, VA_TMS_, MVC, MEP at 10% MVC, Mmax during MVC, Mmax during 10% MVC and Mmax at rest to detect time effects, treatment effects, and time × treatment interaction effects. *Post hoc* tests were done with pairwise comparisons and a Bonferroni correction. When an ANOVA showed a time effect, we made comparisons only for the factor time. When an interaction was shown, we made comparisons for one factor with the other factor fixed and vice versa, i.e., comparisons on the different time point for each treatment and comparison between treatments for each time point.

Retrospective power analysis was applied for the first measurement of VA_TMS_, VA_PNS_, potentiated twitches, MEP and MVC done after the WAnT with sham or iTBS treatment. Power calculations were done for a paired *t*-test, with an effect size represented by the Cohen’s *d* and a statistical significance of 0.05.

## Results

The typical power curve obtained during a WAnT after either iTBS or control in one subject is depicted in Figure 3A. A two-tailed paired *t*-test demonstrated higher peak power in the WAnT after iTBS compared to the controls (961 SE 51 W for control and 970 SE 7 W for iTBS, *p* = 0.037; all numbers following SE correspond to the standard error of the mean) as well as a higher max cadence (153 SE 3 rpm for control and 154 SE 3 rpm for iTBS, *p* = 0.04). We found no significant differences in mean power, fatigue index, and time to reach the peak power (see Figure 3B).

Moreover, we could not observe a significant difference in the amplitude of the 10 MEPs measured before, and 1 min after, the treatment irrespective of the treatment itself (108 SE 6% for control and 112 SE 5% for iTBS, *p* = 0.64). However, as depicted in Figures 2A,C, iTBS significantly increased the MEP amplitude by 28% 7 min after the treatment. It must be noted that in this complementary experiment the time-window with increased MEPs corresponds to the period of WAnT testing in the main experiments. It is also remarkable that during this complementary experiment, however, the iTBS treatment had no visible effect in two out of the six subjects tested.

**Figure 4:**
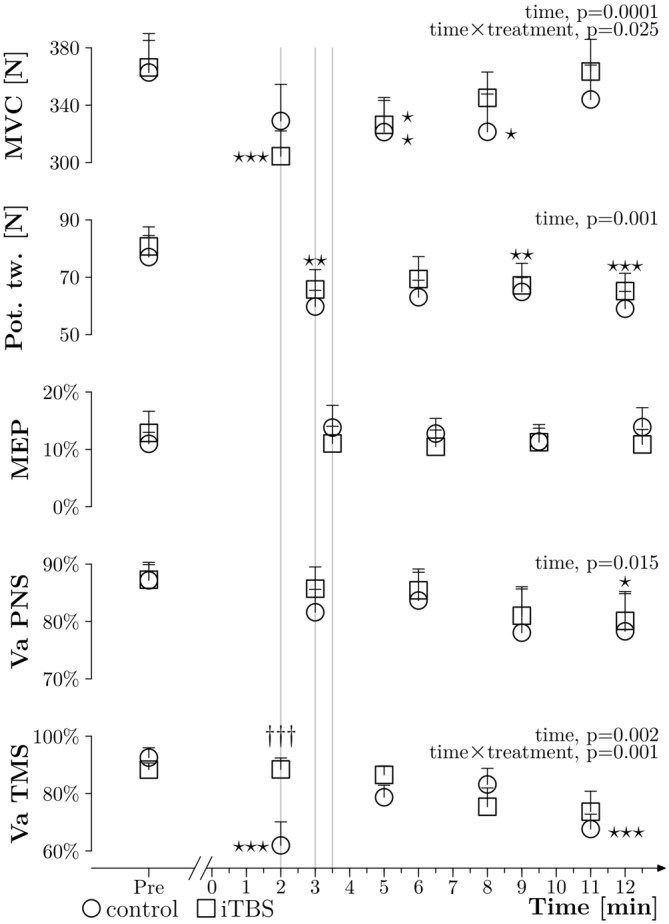
**General results.** Grouped data (*N* = 10) about MVC obtained during VA_TMS_ (in N), amplitude of potentiated twitches at rest (expressed in N), MEPs measured during 10% MVC (expressed in % Mmax), VA_PNS_ and VA_TMS_ plotted against time, before (PRE) and after the WAnT (0 min corresponds to the end of the WAnT), with the control treatment (circles) and with the iTBS treatment (squares); thin lines represent the standard error of the mean. The three thin gray vertical lines highlight differences in timing between measurements. Two way ANOVAs within subjects were applied and significant results are described on the right side of each curve. Stars correspond to a significant difference (one star: *p* < 0.05, two stars: *p* < 0.01 and three stars: *p* < 0.001) related to time effect. Daggers represent a treatment difference for a time point (*p* < 0.001).

As presented in Figure 4, the ANOVAs showed time effects, but no treatment and time × treatment effects, for the potentiated twitches elicited with PNS at rest (*p* = 0.001, *p* = 0.261 and *p* = 0.897, respectively), as well as for VA_PNS_ (*p* = 0.015, *p* = 0.462 and *p* = 0.906, respectively). No significant effects were seen for the MEP amplitudes (*p* = 0.938, *p* = 0.591 and *p* = 0.432). For MVC measured during VA_TMS_ we observed a time and time × treatment effect (*p* ≤ 0.001 and *p* = 0.025, respectively) but no treatment effect (*p* = 0.72). *Post hoc* tests showed: (i) a difference between PRE and Post 2 (*p* = 0.019), PRE and Post 3 (*p* = 0.02) for the control treatment. (ii) A difference between PRE and Post 1 (*p* ≤ 0.001), PRE and Post 2 (*p* = 0.029), Post 1 and Post 3 (*p* = 0.025) and Post 1 and Post 4 (*p* ≤ 0.001) for the iTBS treatment. For VA_TMS_ we observed a time effect as well as a time × treatment effect (*p* = 0.002 and *p* = 0.001, respectively), but no effect for treatment alone (*p* = 0.286). *Post hoc* tests showed differences between PRE and Post 1 (*p* ≤ 0.001), PRE and Post 4 (*p* ≤ 0.001) and between Post 1 and Post 3 (*p* = 0.008) for the control treatment. Moreover, there was a difference between treatments at PRE 1 (*p* ≤ 0.001).

**Figure 5:**
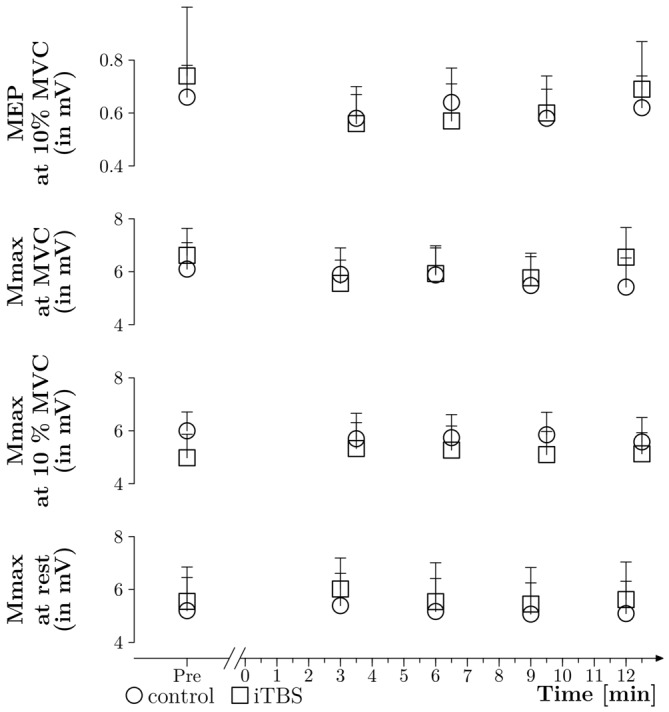
**Mmax and MEP absolute values.** Absolute amplitudes of MEPs were measured during 10% MVC contraction. The TMS intensity was set in order to obtain MEP equal to 10–20% of Mmax before the WAnT and then kept constant throughout the experiment. Mmax was elicited during 10% MVC contraction, at rest in the potentiated muscle (obtained during VA_PNS_ procedure) and during MVC (also obtained during VA_PNS_ procedure). All values are expressed in mV and the thin lines correspond to the standard error of the mean.

As displayed in Figure 5, there were no effects of time and treatment on Mmax measured during MVC (*p* = 0.06, 0.10 and 0.41 for time, treatment and time × treatment respectively), during 10% MVC (*p* = 0.98, 0.84 and 0.50) and at rest (*p* = 0.11, 0.49 and 0.96).

Retrospective power analysis applied on the first measurement done after the WAnT and comparing the values obtained with sham or iTBS treatment were done for VA_TMS_ (Cohen’s *d* = 1.29, power = 0.95), VA_PNS_ (Cohen’s *d* = 0.32, power = 0.15), potentiated twitch at rest (Cohen’s *d* = 0.15, power = 0.07), MEP (Cohen’s *d* = 0.25, power = 0.11) and MVC (Cohen’s *d* = 0.35, power = 0.17).

## Discussion

The present study has shown for the first time that iTBS, applied 5 min prior to a WAnT, could improve peak power and maximum pedalling cadence, and could suppress the subsequent supraspinal fatigue normally observed in the quadriceps muscles in healthy subjects during a high intensity sport specific task. However, the suppression of supraspinal fatigue was not accompanied by a higher performance during the later phases of the task.

### The Effect of iTBS on WAnT

The increase of peak power observed after the iTBS treatment is statistically significant but very small (1% increase). It has to be acknowledged that this difference between the treatments could come from a placebo effect or even a statistical alpha error, and seems to be too small to be of applied relevance. However, when taking into account the context of the task, this result seems plausible and relevant. Firstly, an increase in power was seen in 7 out of the 10 subjects (2 subjects experienced a decrease in power and one had no modifications). Secondly, when considering the improvements in the peak power seen between the three familiarization sessions, an increase of 1% is performance relevant: an increase of 2.1% between session 1 and session 2 (from 919.8 W to 944.8 W), and an increase of 0.9% between session 2 and session 3 (from 944.8 W to 953.4 W). Thus, the iTBS protocol used in the present study seems to be potentially relevant in increasing performance in a maximal anaerobic cycling exercise. We believe that further research in this direction and especially toward the optimization of training throughout a whole sport season could bring new interesting insights in the actual training concepts.

Interestingly, the differences in performance seem to be restricted to peak power and maximal cadence, as we were not able to show any differences in mean power or in the fatigue index of a WAnT. This rather specific effect may be explained by an increased neural drive induced by iTBS. An enhanced neural drive, mainly because of an increased excitability of the excitatory synaptic inputs to pyramidal neurones after iTBS (Di Lazzaro et al., 2008) might modify the efficiency of motor neuronal recruitment (Kernell and Hultborn, 1990), which would allow an easier recruitment of large motoneurons. This enhanced neural drive may also increase the frequency of discharge of motoneurons (Desmedt and Godaux, 1977), and consequently may increase the force production of the discharging motor units (Mrówczyński et al., 2015). Both mechanisms would increase muscular power and the pedalling cadence at the beginning of the maximal cycling task, but do not necessarily affect power over the entire period of the task or the following MVCs.

An alternative explanation for the enhancement of peak power could come from an increased motor learning effect due to the iTBS protocol (Teo et al., 2011). Indeed, during the warm up, which was done right after the iTBS treatment, the subjects had to increase their cadence on two occasions, as quickly as possible. However, in our case, this hypothesis seems unlikely due to the three previous familiarization sessions, which were made to specifically reduce a potential bias arising from motor learning.

Moreover, it seems unlikely that the suppression of supraspinal fatigue with the iTBS treatment directly influenced the peak power and the maximal cadence, as it has been reported that during a maximal 5 s cycling sprint, the EMG activity of the muscles mainly responsible for overall power in the task (such as the VL) doesn’t change, which suggests an as yet unaltered VA at the time you reach peak power (Hautier et al., 2000).

### Suppression of Supraspinal Fatigue Without Changes in Fatigue Related Performance

In the present study supraspinal fatigue after iTBS was considerably lower compared to control. It must be noted that our control measurements are in line with previous results. Indeed, in our study, VA_TMS_ went from 92% to 61% after the WAnT, and in the study of Fernandez-del-Olmo et al. (2013), VA_TMS_ went from 89% to 62%. This high reproducibility of control VA_TMS_ measurements is a strong indicator that iTBS removed a part of the supraspinal fatigue (88% after the iTBS protocol vs. 61% after the control protocol).

Given this significant difference in supraspinal fatigue the question arises: why couldn’t we observe any changes in fatigue related performance? A less-impaired VA should, in theory, allow higher muscle force and power throughout the fatiguing task. Thus, we would expect a higher mean power during the WAnT, as well as a higher MVC after the WAnT, which we were not able to show in the present study. Three possible explanations arise.

Firstly, as we did not measure VA_TMS_ during the WAnT, it is possible that iTBS did not affect the reduction of VA_TMS_ during the task, but only affected its recovery after the end of the task. This would explain the lack of any fatigue related performance differences between the iTBS and control treatments and the increased VA_TMS_ 2 min after the end of the WAnT. However, this hypothesis does not explain the presence of a low MVC despite a high VA_TMS_.

Secondly, it could be that the systemic stress and the peripheral fatigue induced by the WAnT masked any potential beneficial effects of a less-impaired VA_TMS_ on fatigue related performance. Indeed, the overall performance during MVC is dependent of both, VA and the ability of the muscle to produce force. The weakest link of the chain then may limit overall performance to the greatest extent. Hence, the result of significantly lower MVC after the WAnT despite high VA after the iTBS treatment in comparison with control may be explained by peripheral fatigue limiting the force output during MVC and presumably also during the WAnT; perhaps as a sub-product of the greater performance in the WAnT induced by real iTBS. This assumption could also explain the difference in the results of our study and the study of Benwell et al. (2006) where the authors didn’t report an increase in force after rTMS, but rather reported a decreased rate of force loss. Indeed, the WAnT is a whole body power exercise and induces a very large amount of systemic stress (Baker et al., 2002; Coppin et al., 2012), whereas a pinch grip is more of a strength task that requires the use of only a few small muscles with a negligible systemic impact. This implies that the limitation of the performance and the failure of the task may not be induced by the same components of fatigue, or at least not in the same proportions, and that the excitability of motor structures could be affected differently (for review, see Gandevia, 2001; Gruet et al., 2013).

Thirdly, central networks that contribute to the overall motor drive but are not located within the corticospinal tract and cannot be assessed with VA_TMS_, may also impair MVC.

### Discrepancies Between VA_TMS_ and VA_PNS_ Measurements

Although it is difficult to make direct comparisons between VA_TMS_ and VA_PNS_ (Todd et al., 2003), supraspinal fatigue is a subset of central fatigue, and thus, after exercise, the decrease of VA_TMS_ should, in theory, not be independent of the decrease of VA_PNS_ (Taylor et al., 2006). However, in our study, after the WAnT, VA_TMS_ was impaired but not VA_PNS,_ and the iTBS treatment significantly influenced VA_TMS_ but not VA_PNS._ In our opinion, the discrepancies between the VA_TMS_ and VA_PNS_ are mainly related to the timing of the measurements in the present study. Indeed, it is well known that recovery from central fatigue in the quadriceps can be very fast (Gruet et al., 2014). Assuming a steep recovery course for central fatigue after the WAnT, it seems possible that we were able to measure a significant lower VA_TMS_ 2 min after the end of the WAnT but failed to measure a reduced VA_PNS_ 3 min after (see Figure 3). It must be noted that we decided to separate VA_TMS_ and VA_PNS_ measurements by 1 min in order to limit the influence of the first measurement on the second one. Indeed, we were not sure that many subjects could truly reach two times MVC without a break of 1 min, due to the acute stress induced by the WAnT.

### Limitations of the Study

Based on the relevant literature, it is rather unusual to deliver an iTBS treatment with the stimulation coil placed over the cortical leg motor area of both hemispheres. We were able to show in an additional experiment (see Figure 2A) that effects on MEPs were similar compared to results in previous studies that placed the coil over the hot-spot of one hemisphere (Huang et al., 2005). Nevertheless, its effects on the neuronal networks regulating the motor output of both sides, and on the interaction of both hemispheres are not known. Another problematic point regarding the iTBS treatment lies in the fact that the control treatment (TMS at 2% MSO) and the real iTBS treatment could obviously be discerned by the subjects. To compensate this issue, the subjects were unaware of the existence of a real treatment and a placebo treatment, and thus not aware of the fact that one treatment only may have a physiological effect. Nevertheless, it cannot be excluded that the difference in peak power between the two groups may be influenced more by a placebo effect rather than by a physiological effect.

The other main limitation comes from the delay of measurements of VA_PNS_ as addressed in the section above. Indeed, with such a long latency, it is probable that a considerable part of central fatigue which exists right after the end of the task can’t be observed anymore due to a fast recovery already 2 min later (Gruet et al., 2014). To reduce the time delay between the end of the fatiguing task and the VA_TMS_ and VA_PNS_ measurements we suggest to include only participants with a very good physical condition who are able to compensate the discomfort and stress and immediately perform at there maximum very shortly after the end of the fatiguing exercise.

## Conclusion

We have shown increased peak power and maximum pedalling cadence during a WAnT, and increased VA measured with TMS, which could be interpreted as a suppression of supraspinal fatigue after an iTBS treatment. However, variables related to the performance over the entire workout period were not affected despite a significant suppression of supraspinal fatigue. This work can be seen as a first indication that non-invasive brain stimulation methods could have a relevancy regarding the optimization of the performance in sport specific tasks.

## Funding

This study was conducted without any external funding.

## Author Contributions

L-SG and MG designed the study. L-SG, PT and RB collected and analyzed the data. L-SG interpreted the data. L-SG and MG drafted the manuscript.

## Conflict of Interest Statement

The authors declare that the research was conducted in the absence of any commercial or financial relationships that could be construed as a potential conflict of interest.

## Abbreviations

iTBS: intermittent theta burst
MEP: motor evoked potential
MSO: maximal stimulator output
MVC: maximal voluntary contraction
PNS: peripheral nerve stimulation
rTMS: repetitive transcranial magnetic stimulation
TMS: transcranial magnetic stimulation
VL: vastus lateralis
VA: voluntary activation
VA_TMS_: voluntary activation estimated with transcranial magnetic stimulation
VA_PNS_: voluntary activation estimated with peripheral nerve stimulation
WAnT: Wingate Anaerobic Test.
